# Developmental dynamics of emotional dysregulation among sexually abused adolescents: an embedded mixed-methods study

**DOI:** 10.3389/fpsyg.2026.1844735

**Published:** 2026-07-15

**Authors:** Shreya Kulshreshtha, Sujata Satapathy, Renu Sharma, Rajesh Sagar, Reeta Mahey

**Affiliations:** 1 Department of Psychiatry, All India Institute of Medical Sciences New Delhi, New Delhi, India; 2 Department of Obstetrics and Gynaecology, All India Institute of Medical Sciences New Delhi, New Delhi, India

**Keywords:** adolescent sexual abuse, developmental emotional regulation, embedded mixed method, emotional dysregulation, mixed methods study, post traumatic stress disorder, Depression, anxiety

## Abstract

**Introduction:**

Adolescence is characterized by heightened emotional reactivity and rapid maturation of emotion regulation (ER) capacities. Sexual abuse during adolescence disrupts normal ER development. Adolescents often struggle to modulate intrusive memories, hypervigilance, and intense affect following sexual abuse, resulting in increased distress and maladaptive coping strategies, including non-suicidal self-injury.

**Methods:**

Using an embedded mixed-methods design, emotional dysregulation and mental health outcomes among sexually abused adolescents were examined. Three focused group discussions with 18 professionals and 43 key interviews with 25 adolescents (14 with penetrative abuse, including 1 gang rape case, and 11 with non-penetrative abuse) and 18 parents/caregivers (*N* = 61) were conducted at child-care institutions and hospital settings. Thematic frequency analysis resulted in 572 codes, 38 subthemes/subdomains, and 6 themes/domains across the three sample groups. Multi-informant perspectives and multiple settings were considered to draw more generalizable conclusions. Screening measures, namely the Patient Health Questionnaire-9 (PHQ-9), Screen for Child Anxiety Related Emotional Disorders (SCARED), and the Children’s Revised Impact of Event Scale (CRIES-13), were used. Qualitative data focused on emotional impact, triggers, relational difficulties, academic consequences, and coping strategies, while quantitative data assessed depressive, anxiety, and post-traumatic stress symptoms.

**Results:**

Across all groups, themes converged on persistent emotional pain, somatic and affective reactivity, hypervigilance, mistrust in relationships, academic decline, and self-harm. Adolescents consistently reported profound emotional dysregulation, including fear, persistent crying spells, anger outbursts, and social withdrawal. Parents highlighted mood volatility, anger, withdrawal, and emotional numbing, whereas professionals identified chronic dysregulation and attachment ruptures as enduring sequelae of abuse. All adolescents scored above the cutoff on the PHQ-9, while 14 scored above the cutoff on the SCARED, and 13 on the CRIES-13.

**Discussion:**

The effects of sexual abuse type, gender differences, parental support, and the obtained themes are discussed in the context of impact severity and ER.

## Introduction

Adolescence is a sensitive developmental period marked by rapid, normative changes in the physiological bases of emotions, emotional reactivity, and regulatory capacities, making it a pivotal stage during which emotion regulation (ER) skills are learned and consolidated (Silvers, 2022). ER refers to the process of managing the occurrence, duration, and intensity of emotionally arousing stimuli/experiences and the psychophysiological–behavioral reactions to those experiences (Morris et al., 2017). Thus, ER essentially involves the capacity to monitor, evaluate, and modulate emotional arousal in ways that are adaptive to an individual’s goals, context, and social demands. ER is fundamental to both psychological wellbeing and social competence, forming a core foundation for navigating the diverse emotional experiences that shape human life (Kupferberg and Hasler, 2023).

Thompson (2008) asserted that during adolescence, individuals begin to master the influence of emotions and related regulatory processes by experiencing, assessing, and expressing emotions within situational contexts and subsequently evaluating the outcomes of those emotional expressions. The Emotion Regulation Model (Gross, 1999) postulated that disruptions can occur at multiple points due to the immaturity of emotion regulatory processes during this developmental period. Sexual abuse during adolescence significantly compromises these regulatory processes, as trauma-related cues often trigger intrusive memories, hypervigilance, and overwhelming emotions that adolescents struggle to modulate. Consequently, survivors frequently experience persistent dysregulation, including intense fear, shame, anger, dissociation, and somatic distress, reflecting both the neurobiological impact of trauma and internalizing mental health problems (IMHPs). In fact, sexual abuse trauma can exert a direct effect on IMHPs, with emotional dysregulation serving as a key mediating mechanism. The kindling hypothesis and sensitization framework explain that trauma heightens emotional sensitivity and increases vulnerability to subsequent stressors (Rauschenberg et al., 2017). Even relatively minor adverse events may trigger disproportionately intense emotional responses in individuals with a history of trauma. Trauma exposure may also result in the accumulation of unprocessed emotions, thereby undermining the development of effective ER strategies. Consequently, adolescents who have experienced sexual abuse may avoid confronting and processing distressing emotional experiences, increasing their risk for internalizing psychiatric symptoms. The reliance on avoidance strategies to manage abuse-related distress reflects disruptions in self-capacities related to ER. As a result, adolescents often struggle to identify, process, and integrate distressing emotions, frequently resorting to emotional suppression, a core feature of emotional dysregulation (Chung and Chen, 2021).

As approximately one in eight adolescent girls experience rape or sexual abuse, with a significant increase reported between 14 and 17 years of age (UNICEF, 2024), and one in six experience intimate partner violence, including sexual violence, before the age of 18 years (Sardinha et al., 2024), examining the relationship between sexual abuse and adolescents’ ER experiences is important. Although evidence regarding the effects of sexual abuse during adolescence on mental health remains limited, as most studies have relied on retrospective accounts of childhood sexual violence among adults, global research has consistently demonstrated that sexual abuse is strongly associated with poor ER. Previous studies further suggest that difficulties in ER constitute a plausible mechanism through which maltreatment increases vulnerability to revictimization, emotional maladjustment, and later psychopathology (Ion et al., 2023). This issue becomes particularly concerning in contexts where stigma and a culture of silence surrounding sexual abuse contribute to delayed disclosure and reduced access to timely support. Such delays may intensify abuse-related distress, hinder the development of adaptive ER strategies, and increase reliance on maladaptive cognitive coping strategies such as suppression, avoidance, and self-blame, as well as behavioral responses, including suicidal and non-suicidal self-injury (NSSI). Although the only mixed-methods study on ER and mental health among adult men explored ER among individuals with histories of childhood sexual abuse, it did not focus on adolescents or their lived experiences and was therefore vulnerable to recall bias (Snow et al., 2022). Similarly, a UK population-based longitudinal study that analyzed retrospective data also reported higher IMHPs among adolescents with histories of sexual violence (Bentivegna and Patalay, 2022). Indian research has similarly documented this pattern (Bhola and Manjula, 2014) and identified childhood sexual abuse as a strong correlate of NSSI behaviors in both clinical and community samples. Recent studies have also highlighted the mediating role of emotional regulation in the relationship between childhood trauma (although it remains unclear whether sexual abuse was specifically included, despite the Childhood Trauma Questionnaire measuring sexual abuse), emotional dysregulation, and IMHPs among adolescents (Sharma et al., 2026). Previous studies either examined cross-sectional associations or used retrospective recollections of the sexual abuse experience and their subsequent perceived effects on mental health during adulthood; hence, they may not adequately depict the true dynamics of ER following sexual abuse during adolescence. Moreover, culture could play an important role in the dynamics of ER subsequent to sexual abuse. Although conducted with a very small sample, the only identified study on sexual abuse among adolescents in India (Deb et al., 2016) reported the most common form of sexual abuse as “touched or looked at private parts” of the victims (53.2%), followed by “made adolescents feel bad/uncomfortable by speaking or showing them adult pictures” (38.3%), “asked or forced adolescents to touch or look at perpetrator’s private parts” (17.0%), and “forced adolescents to have sexual intercourse” (10.6%). However, it was a prevalence study. Therefore, studying the relationship between sexual abuse, ER, and other correlates through a mixed-methods study will shed light on the lived experiences of adolescents, parents/caregivers’ perspectives, and professionals’ perspectives on the subject. This, in turn, has important policy and public health implications for designing preventive strategies, as sexual abuse in childhood/adolescence increases an individual’s likelihood of experiencing future sexual or physical abuse and may also increase the chances of becoming a perpetrator later in life (Smith et al., 2017). Thus, the study focused on emotional regulation/dysregulation among sexually abused adolescents as the core construct and on specific emotions related to aspects of psychopathology (e.g., depression, anxiety, and post-traumatic stress disorder [PTSD]).

## Methodology

### Study design

An embedded (nested) mixed-methods design was followed. The qualitative data were collected first, and subsequently, quantitative data were collected to supplement the qualitative findings. The qualitative aspect was provided greater methodological weight due to the exploratory nature of emotional dysregulation following adolescent sexual abuse. In-depth interviews with adolescents, caregivers, and professionals provided rich experiential data on the emotional consequences of sexual abuse. The quantitative data focused on three screening tools, namely the Patient Health Questionnaire-9 (PHQ-9; depressive emotions), Screen for Child Anxiety Related Emotional Disorders (SCARED; anxious emotions), and the Children’s Revised Impact of Event Scale (CRIES-13; trauma-related emotions).

### Participant selection criteria

(1) Adolescents were eligible to participate if they had a history of penetrative or non-penetrative sexual abuse within the past year, were male and female adolescents aged 12–18 years, and were registered as a legal case under the Indian legal framework (Protection of Children from Sexual Offences [POCSO] Act, 2012). Written informed consent from the legal authority and assent from participants for a detailed interview were obtained. (2) Parents/caregivers were included if they were the parents/caregivers of adolescents who had experienced a confirmed episode of sexual abuse, if the father, mother, or both were available, and if they were willing to participate in a detailed interview. (3) Professionals were included if they had experience working with adolescent sexual abuse cases, were actively involved in managing such cases, and were willing to participate in a detailed interview.

### Setting

To enhance data variability, adolescents from the government-owned child-care institutions (CCIs), schools, and the psychiatry outpatient department (OPD) of a large tertiary care hospital were recruited.

### Sampling

A purposeful sampling strategy with a parallel/concurrent sampling technique was used, whereby different samples are drawn from the same population at the same time to triangulate results. As the primary qualitative (QUAL) method dominated, a smaller secondary method was “embedded” within it to provide supportive information, often occurring simultaneously. The primary method dictated the main sample size, while the secondary method provided a supportive, in-depth context. Sample size was based on the principle of saturation and pragmatic considerations in concurrence with relevant literature (Britten, 1995; Morse, 2000; Ritchie and Lewis, 2003; Hammersley, 2015; Malterud et al., 2016) and on acceptable and necessary sample size disparity.

### Sample size

For the qualitative study, the sample consisted of 25 adolescents (females = 21; males = 4), recruited from schools (*n* = 3), CCIs (*n* = 18), and psychiatry OPD settings (*n* = 4), 18 parents/caregivers of sexually abused adolescents, and 18 professionals (clinical psychologists, child psychologists, psychiatrists, and gynecologists) working with adolescents with histories of sexual abuse. However, for the quantitative part of the study, 15 adolescents (7 from the original 25 participants and 8 newly recruited adolescents) participated, of whom 8 were adolescent–parent dyads from the original sample. Moreover, 18 adolescents from the original qualitative sample were unable to participate in the quantitative assessment for various reasons (inability to read and write, thereby preventing self-reporting = 6; refusal to consent = 2; relocation from the child-care institution = 10). As most adolescents were recruited from CCIs, only 18 available and consented parents were included.

### Tools for data collection

1. Socio-demographics questionnaire
  A brief socio-demographic questionnaire was used to collect background information about the participants. This included details such as age, gender, educational status, and key abuse-related characteristics, including the number of abuse episodes, whether the abuse was intrafamilial or extrafamilial, and whether it involved penetrative or non-penetrative acts in case of adolescents. For caregivers, demographic information such as age, gender, and relationship to the adolescent was recorded. For professionals, age and gender details were collected to describe the sample.
2. Focus group discussion (FGD) and key interview (KI) guide
  Separate interview guides were developed by the authors for adolescents, caregivers, and professionals. The questions were developed through the identification of relevant issues based on the literature and clinical experience, followed by brainstorming of subdomains relevant for inclusion in the FGD/KI guides for the different target groups. The FGD/KI guide contained open-ended questions on the importance and experience of emotions during adolescence, the bidirectional relationship between emotional experiences, stressors, and abuse, triggers for emotional dysregulation, common reactions to extreme emotions, trust in relationships, academic and identity-related consequences, self-harm, aggression, other behavioral consequences, and adaptive and maladaptive coping strategies employed.
  The guide was reviewed by two external clinical psychologists who were not part of the guide development team. The pilot testing was conducted with 2–3 participants from each group. The guide was further revised based on participant feedback from each category. The final interview guide had 11 questions for adolescents, 10 for parents, and 4 for professionals. The context of the questions was restricted to sexual abuse and the consequent emotional experiences.
  Each KI lasted 45–60 min across all three sample groups, whereas FGDs lasted 60–90 min. The interviews were audio-recorded (following written informed consent) and transcribed verbatim for professionals and parents. Owing to ethical considerations prohibiting audio recording at CCIs, real-time verbatim field notes were manually recorded during interviews with adolescents.
3. Screen for child anxiety related emotional disorders (SCARED) (Birmaher et al., 1997)
  The tool is used to screen for anxiety disorders, including generalized anxiety, separation anxiety, social phobia, panic, and school refusal, among children aged 8–18 years. The reliability coefficient typically ranges from *α* = 0.74 to 0.93, and the test–retest reliability correlation coefficients range from 0.70 to 0.90. The tool demonstrates good discriminant validity. The cutoff for the scale is ≥25.
4. Patient health questionnaire-9 (PHQ-9) (Kroenke et al., 2001)
  The PHQ-19 was used to assess self-reported depressive symptom severity according to DSM-5 criteria. It has a sensitivity and a specificity of 88% for major depressive disorder. The Cronbach’s alpha of the PHQ-9 is 0.89, indicating excellent reliability. A cutoff score of ≥10 was considered indicative of depression among adolescents (Johnson et al., 2002).
5. Children’s revised impact of event scale (CRIES-13; Perrin et al., 2005)
  The Cries-13 was used to screen for PTSD and assess three core symptom clusters of intrusion, avoidance, and arousal. The CRIES-13 showed a good Cronbach’s *α*, typically ≥0.80. In two clinical samples, the CRIES-13 demonstrated sensitivity values of 0.91 and 0.86 and specificity values of 0.65 and 0.73, respectively. The cutoff for the scale is ≥30.

### Data analysis

Qualitative data were analyzed using thematic frequency analysis (Braun and Clarke, 2024). The analytical process began with inductive coding, wherein initial codes were derived directly from participants’ narratives of adolescents, caregivers, and professionals. These codes were subsequently organized into conceptual groupings, allowing for the identification of broader patterns across the three informant datasets. To ensure depth and rigor, themes were refined through an iterative review process involving continuous movement between the coded extracts and the entire dataset. The frequency of each code across all participants was calculated to understand the relative prominence of each code within and across groups.

Descriptive statistical analysis was conducted for the quantitative data. Additionally, each transcript was analyzed in relation to the adolescent’s scores on the scales.

### Ethical considerations

- Inclusion was restricted to legally registered adolescent sexual abuse cases under the Indian legal framework, the POCSO Act, 2012, recruited from schools and CCIs. Prior permission from both settings was obtained.
- Informed Consent Process: All participants were provided with a Participant Information Sheet detailing the study’s aim, rationale, and benefits, while explicitly assuring the confidentiality of personal data and participants’ autonomy to withdraw at any point during the interview or group discussion.
- Consent and Assent: In accordance with ethical guidelines for minors, written parental consent and adolescent assent were obtained before participation.
- Participant Safety and Support: Data collection followed a trauma-informed approach to interviewing, with immediate crisis intervention support available throughout the sessions to mitigate potential distress resulting from trauma recall and reliving.

## Results

Table 1 presents the demographic characteristics of the three participant groups included in this study. The preponderance of female participants was observed in both the adolescent and professional groups. Approximately 72% of adolescents were in their late adolescence, resulting in a mean age of 15.5 years. Most adolescents had experienced multiple episodes of sexual abuse, and 60% had experienced penetrative sexual abuse. While analyzing the demographic (mean age and gender) and abuse-related characteristics (type of abuse, number of abuse episodes, intrafamilial/extrafamilial abuse, and mean duration between the abuse and disclosure) of the total sample, data from both the qualitative and quantitative samples were combined after excluding the overlapping participants (*n* = 7). Thus, data from 33 adolescents were included in the analysis.

**Table 1:** Demographic characteristics of adolescents, caregivers, and professionals.

Parameters	Adolescents n1 = 25 (%) qualitative	Adolescents n2 = 15 (%) quantitative	Total adolescents *N* = 33 (%)	Parents *N* = 18 (%)	Professionals *N* = 18 (%)
Age (years): mean ± SD Early adolescence (12–14 years) Late adolescence (15–18 years)	15.5 ± 0.69 7 (28) 18 (72)	15.8 ± 2.1 5 (33.3) 10 (66.6)	15.6 ± 1.9 10 (30.3) 23 (69.6)	40.9 ± 4.5	32.0 ± 4
Females Males	21 (84) 4 (16)	13 (86.6) 2 (13.3)	27 (81.8) 6 (18.1)	M = 10 (55.6) *F* = 8 (44.44)	17 (94.4) 1(5.6)
Single episode Multiple episodes	5 (20) 20 (80)	3 (20) 12 (80)	7 (21.2) 26 (78.7)	–	–
Intrafamilial Extrafamilial	11(44) 14 (56)	9 (60) 6 (40)	17 (51.5) 16 (48.4)	–	–
PSA NPSA	15 (60) 10 (40)	8 (53.3) 7 (46.6)	18 (54.5) 15 (45.4)	–	–
Mean duration since abuse occurrence (months)	5.3	6.1	6	–	–

PSA, penetrative sexual abuse; NPSA, non-penetrative sexual abuse; M, mother; F, father.

### Qualitative study

As presented in Table 2, a total of 572 codes were generated. The codes were grouped and organized into 38 subthemes, which were further categorized according to relevant psychological constructs and represented under six themes related to emotion regulation: (1) extreme emotions: affective responses; (2) extreme emotions: fear and anxious emotions; (3) emotion regulation–dysregulation paradigm: anger as a common behavioral manifestation; (4) emotion regulation–dysregulation paradigm: maladaptive behavioral manifestations; (5) emotion dysregulation: disturbed internal worldview; (6) emotion dysregulation: disturbed external worldview.

**Table 2:** Findings from the qualitative study: themes, subthemes, and examples of codes.

S. No.	Themes	Subthemes of emotion regulation	Codes merging into subthemes	Examples of codes related to emotion regulation (A = adolescents, C = caregivers, and P = professionals)	Frequency (%) of codes
1	Basic to extreme emotions: sadness and affective responses	1.1 Interpersonal withdrawal (A) 1.2 Academic avoidance (A) 1.3 Lack of motivation 1.4 Recurrent crying spells (A, C) 1.5 Low mood (A, C) 1.6 Loss of interest and pleasure (A, C, P)	1.1 (Preferring isolation, avoidance of friends) 1.2 (School refusal, reduced academic interest) 1.3 (Reduced motivation, decline in concentration) 1.4 (Crying during conversations, swollen eyes in the morning, sudden crying without clear triggers, crying when alone) 1.5 (Prolonged sadness, emotional numbness, lowered emotional thresholds, depression-like withdrawal, difficulty predicting mood) 1.6 (Withdrawal from creative activities previously enjoyed, withdrawal from hobbies)	Crying during trauma-related conversations (A) Emotional numbness (A) Preferring isolation (A) Refusal to talk about the incident (A) Emotional shutdown when questioned (A) School refusal (C) Swollen eyes in the morning (C) Withdrawal from creative activities previously enjoyed (C) Perceived permanent damage (P) Crying when alone (A, C) Sudden crying without clear triggers (A, C) Prolonged sadness (A, C) Reduced motivation (A, C) Avoidance of friends (A, C) Decline in concentration (A, C) Withdrawal from hobbies (A, P) Reduced academic interest (A, C, P) Lowered emotional thresholds (A, C, P) Depression-like withdrawal (A, C, P) Difficulty predicting mood (A, C, P)	12 (48%) 4 (16%) 3 (12%) 2 (8%) 5 (27.7%) 7 (16.2%) 6 (13.9%) 6 (13.9%) 5 (13.8%) 13 (33.4%) 11 (18%) 10 (23.2%) 9 (20.9%) 9 (36%) 7 (31%) 18 (41.9%) 15 (24.5%) 7 (11.4%) 9 (14.7%) 3 (4.9%) Total codes = 161
2	Basic to extreme emotions: fear and anxiety-related emotions	2.1 Fear during abuse (A) 2.2 Fear of disclosure and retaliation (A, P) 2.3 Persistent anxiety and hyperarousal (A) 2.4 Panic symptoms (A, P) 2.5 Freeze and dissociative responses (A, P) 2.6 Body-based trauma responses (A, C, P) 2.7 Emotion dysregulation without identifiable triggers (A) 2.8 Difficulty being soothed (C) 2.9 Hypervigilance (P)	2.1 (Confusion about what was happening, fear during the incident) 2.2 (Fear of social judgment, fear due to perpetrator’s threats) 2.3 (Fear of being scolded or blamed, ongoing nervousness, persistent bodily tension, heightened alertness in daily settings, flashbacks, nightmares, fear of going out) 2.4 (Panic-like reactions) 2.5 (Feeling frozen or numb, blankness during distress) 2.6 (Racing heart, breathlessness, trembling or shaking, physical fear responses (tension/discomfort) 2.7 (Anxiety without identifiable triggers) 2.8 (Anxiety spirals become overwhelming for caregivers) 2.9 (Avoidance of the place of abuse, perceived danger everywhere, apprehension around men)	Confusion about what was happening (A) Fear of being scolded or blamed (A) Fear of social judgment (A) Fear due to perpetrator’s threats (A) Ongoing nervousness (A) Racing heart (A) Breathlessness (A) Feeling frozen or numb (A) Blankness during distress (A) Persistent bodily tension (A) Anxiety without identifiable triggers (A) Panic-like reactions (A, P) Heightened alertness in daily setting (A, P) Trembling or shaking (A, P) Anxiety spirals become overwhelming for caregivers (C) Physical fear responses (tension/discomfort) (A, P) Flashbacks, nightmares (P, C) Avoidance of the place of abuse (P, C) Fear of going out (P, C) Perceived danger everywhere (P, C) Apprehension around men (P, C) Fear during the incident (A, C, P)	5 (20%) 6 (24%) 4 (16%) 4 (16%) 5 (20%) 6 (24%) 3 (12%) 3 (12%) 3 (12%) 8 (32%) 5 (11.6%) 4 (9.3%) 6 (13.9%) 5 (20%) 9 (20.9%) 5 (27.7%) 8 (18.6%) 6 (16.6%) 6 (16.6%) 4 (11.1%) 10 (27.7%) 14 (22.9%) Total codes = 129
3	Emotion regulation–dysregulation paradigm: anger as a common behavioral manifestation	3.1 Anger toward the perpetrator (A) 3.2 Generalized and increased Irritability (A, C, P) 3.3 Poor anger regulation (A, C, P) 3.4 Rapid mood swings (C, P) 3.5 Increased sensitivity C) 3.6 Long-term impact (P) 3.7 Relapse of symptoms (P) 3.8 Emotional mislabeling (P)	3.1 (Intense anger on seeing or remembering the abuser, urges for retaliation) 3.2 (Low frustration tolerance, disproportionate emotional reactions, irritability) 3.3 (Distress intolerance, breaking or throwing objects, frequent anger outbursts, inability to control anger) 3.4 (Anger episodes followed by crying) 3.5 (Hypersensitivity, startled responses, reactivity to minor triggers, disgust toward touch) 3.6 (Long-term trauma impact, symptoms resurfacing after years) 3.7 (Symptoms reactivated by triggers) 3.8 (All negative feelings perceived as anger, difficulty articulating sadness or loneliness, need probing to uncover underlying emotion)	Intense anger on seeing or remembering abuser (A) Urges for retaliation (A) Anger episodes followed by crying (C) Long-term trauma impact (P) Symptoms resurfacing after years (P) Symptoms reactivated by triggers (P) Distress intolerance (P) Hypersensitivity (P) Startled responses (P) All negative feelings perceived as anger (P) Difficulty articulating sadness or loneliness (P) Need probing to uncover underlying emotion (P) Low frustration tolerance (A, C) Reactivity to minor triggers (A, C) Breaking or throwing objects (A, C) Disproportionate emotional reactions (A, C) Disgust toward touch (A, P) Irritability (C, P) Frequent anger outbursts (A, C, P) Inability to control anger (A, C, P)	9 (36%) 3 (12%) 5 (27.7%) 3 (16.6%) 2 (11.1%) 3 (6.9%) 3 (16.6%) 2 (11.1%) 2 (11.1%) 2 (11.1%) 2 (11.1%) 1 (5.5%) 9 (22.5%) 7 (17.5%) 12 (27.9%) 5 (38.8%) 2 (11.1%) 7 (38.8%) 15 (25.8%) 11 (25.5%) Total codes = 105
4	Emotion regulation–dysregulation paradigm: maladaptive behavioral manifestations	4.1 Avoidant coping (A) 4.2 Externalizing behaviors (A, P) 4.3 Escalated anger episodes (A) 4.4 Aggressive behavior toward others (A) 4.5 Avoidance of trauma discussion (A) 4.6 Self-harm (C, P) 4.7 Desire to escape emotional pain (C) 4.8 Non-disclosure of internal states (C) 4.9 Reduced social interaction (C)	4.1 (Quitting school, increased absenteeism from school) 4.2 (Loss of emotional and physical restraint, breaking objects) 4.3 (Anger episodes become explosive and uncontrolled) 4.4 (Violence with family members) 4.5 (Emotional privacy) 4.6 (Anticipation of harm to self again, self-harm ideation, multiple attempts to harm self by cutting/overdosing, self-harm attempts) 4.7 (Emotional numbing leading to impulsive acts) 4.8 (Avoidance of discussing the traumatic day) 4.9 (Social avoidance)	Social avoidance (A) Anticipation of harm to self again (A) Quitting school (A) Increased absenteeism from school (A) Avoidance of discussing the traumatic day (C) Anger episodes become explosive and uncontrolled (C) Loss of emotional and physical restraint (C) Emotional privacy (C) Emotional numbing leading to impulsive acts (P) Breaking objects (A, C) Self-harm ideation (A, P) Multiple attempts to harm self by cutting/overdosing (C, P) Violence with family members (C, P) Self-harm attempts (A, C, P)	4 (16%) 3 (12%) 3 (12%) 2 (8%) 7 (38.8%) 3 (16.6%) 3 (16.6%) 3 (16.6%) 2 (11.1%) 8 (18.6%) 12 (27.7%) 8 (44.4%) 3 (8.3%) 21 (34.4%) Total codes = 82
5	Emotion dysregulation: disturbed internal worldview	5.1 Emotional helplessness (A) 5.2 Self-Blame and shame (A) 5.3 Emotional overwhelm (A) 5.4 Life-role disruption (P)	5.1 (Feeling powerless, distress over inability to protect oneself) 5.2 (Blaming oneself for the abuse, feeling “dirty” or guilty, negative self-perception) 5.3 (Sense of emotional collapse, feeling mentally overwhelmed, difficulty understanding emotions) 5.4 (Self-peer comparison, guilt for underperformance, feeling “left behind”)	Feeling powerless (A) Distress over inability to protect oneself (A) Sense of emotional collapse (A) Self–peer comparison (P) Guilt for underperformance (P) Feeling “left behind” (P) Blaming oneself for the abuse (A, C) Feeling “dirty” or guilty (A, C) Feeling mentally overwhelmed (A, C) Difficulty understanding emotions (A, P) Negative self-perception (A, C, P)	4 (16%) 3 (12%) 2 (8%) 2 (11.1%) 2 (11.1%) 2 (11.1%) 10 (23.2%) 5 (11.6%) 4 (9.3%) 11 (25.5%) 5 (8.1%) Total codes = 50
6	Emotion dysregulation: disturbed external worldview	6.1 Fear and mistrust in relationships (A, C, P) 6.2 Expectation of betrayal and emotional distance (A, C, P)	6.1 (Attachment insecurity and abandonment fears, fear of intimacy, disgust toward physical touch even from loved ones, relational anxiety, fear of future relationships, mistrust even toward parents, loss of trust in known persons) 6.2 (Expectation of abandonment, emotional distancing from caregivers)	Attachment insecurity and abandonment fears (P) Fear of intimacy (P) Expectation of abandonment (P) Disgust toward physical touch even from loved ones (P) Relational anxiety (A, C) Emotional distancing from caregivers (A, C) Fear of future relationships (A, P) Mistrust even toward parents (P, C) Loss of trust in known persons (A, P, C)	3 (16.6%) 2 (11.1%) 4 (22.2%) 5 (13.8%) 5 (27.75) 6 (13.9%) 6 (13.9%) 2 (5.5%) 12 (19.6%) Total codes = 45

For frequency and percentage analyses of each code, the denominator was selected based on the number of participants within each group who reported the corresponding code.

Out of 572 codes, 120 were from adolescents, whereas 49 and 47 codes were generated from caregivers and professionals, respectively (Table 2). Shared codes between adolescents and caregivers totaled 130, whereas 69 codes were shared between adolescents and professionals. Among caregivers and professionals, 60 codes were common. All three groups shared 97 codes.

### Theme 1: Basic to extreme emotions—affective responses

The maximum number of codes (28.1%) was observed in the domain of basic to extreme emotions: affective responses. The subthemes included interpersonal withdrawal, academic avoidance, lack of motivation, recurrent crying spells, persistent low mood, and loss of interest and pleasure, indicating that intense emotional reactions were central to participants’ narratives. A girl who was abused by her father shared, *“I feel like crying a lot… even while talking on unrelated topics, I suddenly burst into tears. These days I also do not feel like doing much.”* A male victim shared, *“When I used to be sad, I would just keep it inside me. I was terrified of meeting people, so I also stopped going to school as well.”* Parents of a female victim expressed their concern: *“She prefers to be alone, she locks herself in her room and does not sit with us anymore since the incident has happened.”*

### Theme 2: Basic to extreme emotions—fear and anxious emotions

This theme accounted for 20.9% of the codes. This theme included subthemes such as persistent anxiety and hyperarousal, panic and physiological symptoms, dissociative responses, and body-based trauma responses. A female adolescent stated, *“I used to feel helpless that I could not share this with anyone. My body starts shaking and I feel a lot of nervousness and panic sometimes.”*

### Theme 3: Emotion regulation–dysregulation paradigm—anger as a common behavioral manifestation

This theme accounted for 18.3% of the codes and included subthemes such as anger toward the perpetrator, increased irritability, rapid mood swings, and difficulty regulating anger. A parent highlighted, *“When she gets angry, it’s extreme. In her anger, she has also hit me on some occasions; she cannot control it. Her mood spoils very quickly. Somedays she would have swollen eyes in the morning from crying so much.”* Importantly, a child psychologist at a child-care institute shared*, “They would mostly label all negative emotions as anger. When probed further, they may say I was feeling lonely or I felt bad. They lack the emotional vocabulary to express what they are feeling which may increase their suffering.”*

### Theme 4: Emotion regulation–dysregulation paradigm—maladaptive behavioral manifestations

This theme highlighted maladaptive coping responses and included subthemes such as avoidant coping, externalizing behaviors, self-harm attempts, and the desire to escape emotional pain, accounting for 13.8% of the codes. A parent shared, *“She once tried to kill herself by overdosing on the prescribed anxiety medicines.”* A female adolescent victim stated*, “I harm myself by compass as soon as I feel like crying or something reminds me of what happened that day.”*

### Theme 5: Emotion dysregulation—disturbed internal worldview

This theme accounted for 8.7% of the codes and included subthemes such as self-blame and shame, emotional confusion, and life-role disruption. An experienced child psychologist shared, *“Academics suffer… there is so much guilt that they are not able to perform.”*

### Theme 6: Emotion dysregulation—disturbed external worldview

This theme accounted for 8.3% of the codes. The subthemes included fear and mistrust in relationships, betrayal, and attachment rupture. A parent observed, *“She tells us nothing about how she feels.”* A clinical psychologist reported, *“Trust even on their parents erodes… they do not think they will be able to understand. This phenomenon of trust in relationships and attachment security gets further complicated in case of incest.”* A female victim shared, *“I used to like talking to one boy… but I started feeling scared of him too, after what my uncle did to me. I do not think I will ever be able to have a boyfriend or marry.”*

Parents’ reactions toward and management of emotion dysregulation in adolescents by the parents: this included parents’ focus on behavioral manifestation of emotions and attempts to use supportive strategies to manage these. The supportive strategies included providing space to the adolescent, as one parent mentioned, “We leave her alone for a while so she calms down.” Some others expressed active listening and presence as a helpful strategy. Like a father noted, *“I try to listen to what is in his heart and show him the right path.”* Constructive distraction was another strategy, like a mother trying to engage her child in skill-building to calm her mind: *“I put her in typing and knitting classes… Her mind gets engaged there.”* Environmental control strategies included some protective and sometimes restrictive measures like social isolation from relatives and neighbors. They were vigilant on who comes to the house to avoid triggers for the child, like a mother said *“We do not allow people to come to the house anymore, we have stopped meeting with most of our known people.”* One family decided to move their home entirely and emphasized that *“Now we are going to shift from this place, not everyone understands.”* Many parents stopped sending children to school or outside due to fear of them fainting (dissociation) or running away. Like a mother shared, *“we have even stopped sending her to school, fearing she might faint there or get too distressed. Right now, sending her to school feels like a risk.”* The maladaptive responses included physical punishment, taunting, and blaming. In several cases, when the child showed anger or “rebellion,” the parents/relatives (especially fathers) responded with violence: *“When her father got to know about what happened he beat her a lot and scolded her and even her uncle (father’s elder brother) beat her severely.”* At times when the child was dysregulated, some parents responded with taunts, like a mother distressingly reported that, *“her father goes as far as to say that you will not be able to achieve anything in life.”*

### Professionals

**Figure 1:**
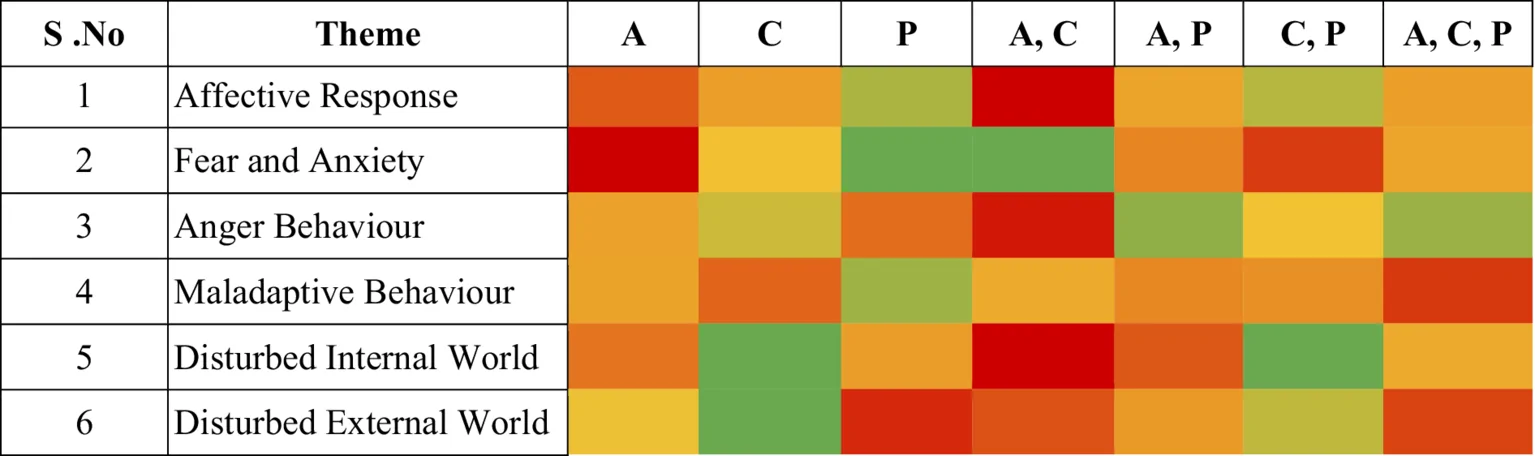
Heatmap of symptom convergence based on percentage of codes. Color shading represents the proportion of coded responses within each theme, with darker shades indicating higher representation of codes and lighter shades indicating lower representation. For the construction of the heatmap, the frequencies of codes were converted to percentages by dividing the number of codes reported by each respondent/group by the total number of codes within the respective thematic category and multiplying by 100. Green: low weightage; yellow/orange: moderate weightage; red: high weightage.

Professionals described patterns of emotional withdrawal, including reduced engagement in academics and hobbies, alongside symptoms resembling depression. Adolescents were perceived to experience heightened anxiety and hypervigilance, reflected in panic-like reactions, trembling, persistent alertness, and fear of everyday environments, often accompanied by trauma-related symptoms such as flashbacks, nightmares, and avoidance of places associated with the abuse. Professionals also noted difficulties in emotional awareness and expression, with adolescents often misinterpreting or masking underlying emotions such as sadness or loneliness as anger. A professional working with institutionalized children highlighted, *“Emotionally, I have noticed the presence of abandonment issues and also a lack of emotional understanding. They would mostly label all negative emotions as anger and when probed further, then they may say that I was feeling lonely or I felt bad. Emotional instability is there. In general, there is a feeling of betrayal so they already have trust issues.”* This emotional dysregulation frequently manifested as irritability, frequent anger outbursts, hypersensitivity to triggers, and low distress tolerance. One professional noted that *“aggression frequently manifests as an emotional response, explaining that adolescents may exhibit verbal or physical aggression, which, when coupled with impulsivity, can function as a maladaptive coping strategy for managing overwhelming emotions.*” In more severe cases, professionals reported self-harm behaviors, suicidal ideation, and impulsive actions. They further observed long-term trauma impacts, including attachment insecurities, fear of intimacy, mistrust of others, and heightened sensitivity to physical touch (Figure 1).

### Quantitative study findings

As revealed in Table 3, the group mean score on CRIES-13 (screening for post-traumatic stress disorder across the domains of avoidance, intrusion, and hyperarousal) was 38.6, whereas the mean scores on SCARED (screening for anxiety disorders across five domains: somatic/panic, general anxiety, separation anxiety, social phobia, and school phobia) and PHQ-9 (screening for depressive symptoms) were 38.20 and 12.80, respectively. All of these were above the cutoff levels, suggesting significant difficulties in ER and emotional functioning among these adolescents. A cutoff score of ≥30 on CRIES-13 indicates clinically significant post-traumatic stress symptoms, whereas scores >25 on SCARED indicate the likely presence of an anxiety disorder, and scores ≥10 on PHQ-9 indicate moderate depressive symptoms. Notably, all adolescents who reported penetrative sexual abuse (PSA) scored above the cutoff scores for PTSD, anxiety, and depression. The Mann–Whitney U test was conducted to examine gender differences across the three scales. Female adolescents had higher PTSD and depression scores than male adolescents, whereas male adolescents had higher anxiety scores than female adolescents. There were no statistically significant gender differences in PTSD and anxiety scales. However, the findings on PHQ-9 indicated statistically significant differences in depressive symptoms between male and female adolescents, with female adolescents reporting higher PHQ-9 scores (𝑧 = 1, *p* < 0.05). The SD among male adolescents on PHQ-9 was 0, showing no variance in scores, which may be attributable to the very small male subsample (*n* = 2) and the limited variability in comparison values. Therefore, the findings should be interpreted with caution due to small and unequal sample sizes.

**Table 3:** Descriptive statistics and Mann–Whitney *U* test findings on quantitative measures among adolescents (*N* = 15).

Measure	Mean (SD)	Male/female mean (SD)	Male vs female: Mann–Whitney *U* test	*p*-value	Adolescents above cutoff (%)
CRIES-13 (PTSD) cutoff = ≥30	38.6 (10.8)	35 ± 7.07/39.15 ± 11.4	*U* = 11.0	0.82	13 (86.7%) PSA = 8, PSA = 5
SCARED (anxiety) cutoff = ≥ 25	38.20 (12.98)	42.0 ± 18.3/37.6 ± 12.8	*U* = 11.5	0.70	14 (93.3%) PSA = 8, NPSA = 6
PHQ-9 (depression) cutoff = ≥10	12.80 (3.45)	9 ± 0/13.3 ± 3.3	*U* = 1.0	0.04	12 (80%) PSA = 8, NPSA = 4

CRIES 13, Children’s Revised Impact of Event Scale (13-item version); SCARED, Screen for Child Anxiety Related Emotional Disorders; PHQ 9, Patients’ Health Questionnaire (9-item version); PSA, penetrative sexual abuse; NPSA, non-penetrative sexual abuse.

## Discussion

Regulation of emotions is a key developmental function influencing both the onset and persistence of emotional and behavioral difficulties in adolescence. Puberty-driven brain changes often cause intense emotions to temporarily overwhelm adolescents’ still-maturing ER systems and frontal lobe functions. This leads to a temporary decrease in regulatory abilities and imbalance in emotional stability during mid-adolescence that typically improves by late adolescence (Tottenham and Galván, 2016; Beauchaine and Cicchetti, 2019; Lynch et al., 2021; Powers et al., 2022). This study utilized a multi-informant approach to explore the phenomenology of emotions and ER following sexual abuse among adolescents (Alves et al., 2024).

Our sample size was comparable to that of a previous study that had adolescents aged 9–19 years (*N* = 48); however, unlike our study, it included only a few adolescents exposed to sexual abuse (Chiasson et al., 2021; Grasser et al., 2025). Similarly, including 18 parents of sexually abused adolescents in our study provided broader caregiver perspectives than those reported in previous qualitative studies (Bayrak and Uzun, 2025; Mauny et al., 2023). Descriptive findings such as the predominance of male perpetrators and disclosure-related difficulties among the adolescents were supported by previous studies. Our finding of 53–60% of PSA was similar to studies reporting 48.9 and 68% (Yüce et al., 2015; Lam, 2015; Mutluer et al., 2018). The findings that 53.5% of adolescent girls and 33.9% of adolescent boys reported experiencing some form of sexual-violence victimization (Ngo et al., 2018) supported our findings.

All findings from the qualitative data were divided into six thematic categories to demonstrate the transition from basic emotions to emotion dysregulation as a developmental continuum: (1) Basic to extreme emotions: sadness/affective response, (2) Basic to extreme emotions: fear and anxiety, (3) Emotion regulation–dysregulation paradigm: anger as a common behavioral manifestation, (4) Emotion regulation–dysregulation paradigm: maladaptive behavioral manifestation, (5) Emotion dysregulation: disturbed internal worldview, and (6) Emotion dysregulation: disturbed external worldview. This also enhanced the significance of the quantitative data indicating possible symptoms of depression, anxiety, and PTSD. Our categorization was based on the Schematic Propositional Analogical Associative Representation Systems (SPAARS) multilevel model (Bower and Dalgleish, 1997; Power and Dalgleish, 2008). We hypothesized that each basic emotion represented a distinct appraisal of the sexual abuse experience among adolescents within a specific context and that the resulting triggered emotions would then be processed by the associative system (automatic emotion processing), the schematic system (processing requiring effortful appraisal), or the propositional system (which indirectly operates via connections with the schematic and associative levels). Emotional dysregulation may therefore result from the coupling of basic emotions or processing levels within the same emotion (e.g., associative and schematic levels). For example, although the combination of sadness and disgust forms the psychological foundation of depression, PTSD stems from fear and disgust (Power and Dalgleish, 1997). However, survivors of sexual abuse may have unique emotional triggers and appraisals because of the interpersonal nature of the abuse, even when they share common PTSD symptoms with other trauma victims (Coyle et al., 2014).

### Extreme emotions: sadness/affective response

All stakeholders reported high levels of sadness, crying spells, withdrawal from social interaction, and general loss of interest, indicating strong alignment with depressive symptomatology. Although adolescents emphasized internal sadness, emotional numbing, and a desire for isolation, parents focused on external manifestations such as observable withdrawal and aloofness, and professionals identified emotional numbing and clinical depression. This highlights how the same emotional state is experienced internally, observed behaviorally, and conceptualized clinically, underscoring the importance of multi-perspective assessment and intervention. Consistent with the literature on informant-discrepancy research, internalizing symptoms are often underreported by caregivers who rely primarily on observable behavior (Achenbach et al., 1987). Although the SPAARS model does not specifically address emotional numbing, it has been identified as a core trauma-related affective disturbance, particularly in individuals exposed to interpersonal trauma such as sexual abuse (Van Der Kolk et al., 2005; Coyle et al., 2014). One concern is that when parents’ primary assessment of the impacts of sexual abuse on adolescents focuses on observable behaviors (e.g., withdrawal/reduced engagement), commonly occurring emotional numbing may be misinterpreted as disengagement or oppositionality, increasing the risk of under-recognition, negative parental reactions, and inadequate treatment. In such cases, depressive symptoms may remain unnoticed, and emotional numbness or internal despair may remain unaddressed. As sexually abused adolescents with internalizing distress generally remain well-behaved, they often do not meet the functional impairment threshold as perceived by adults, which may lead to significant delays in care and an increased risk of clinical depression (Lubit et al., 2003; Kolko et al., 2010). The finding that 80% of adolescents were screened positive on the PHQ-9 scale supplemented the qualitative findings on the expression of extreme sadness by adolescents and parents. Although almost half of the sample comprised adolescents in both samples, there was matching of.

### Extreme emotions: fear and anxious emotions

Parents reiterated adolescents’ experience of autonomic responses of anxiety (shivering, fear, and nervousness), whereas professionals framed fear within hypervigilance and PTSD symptoms. The findings of 93.3% of adolescents scoring above the cutoff on SCARED also corroborate the qualitative findings. Research supports these findings, showing that sexual abuse in childhood can disrupt the functioning of the HPA axis, limbic system, and stress response system, leading to persistent hyperarousal, heightened physiological anxiety responses, and increased vigilance to perceived threat (De Bellis et al., 1994; De Bellis et al., 2011). Considering Dalgleish and Power’s (2004) SPAARS model, our study confirmed that emotions related to PTSD primarily consisted of fear and anxiety as emotion-specific components and hypervigilance as a non-specific component. Disgust as part of the emotion-specific PTSD component will be discussed as part of behavioral manifestation in the next sections.

The primary findings reveal a significant convergence of all stakeholders on the theme of expression of emotion through externalized behavioral issues such as anger and irritability following sexual abuse. Although original studies on this topic among adolescents are limited, case studies on young children report acting out and anger as the most prominent emotional-behavioral consequences of sexual abuse (Al Odhayani et al., 2013). Additionally, trauma is associated with heightened irritability in adolescents and is therefore conceptualized as a transdiagnostic psychiatric phenotype marked by an increased tendency toward anger compared with peers (Grasser et al., 2025). Although their study characteristics differed from ours—for example, irritability was measured using a structured tool, they included a control group, and the sample size for adolescents with sexual abuse histories was not specified—their findings on trauma and irritability can be extrapolated to explain our findings on post-sexual abuse irritability. Furthermore, our findings also highlighted the internalized aspects of the effects of sexual abuse on emotional dysregulation due to attachment rupture, trust betrayal, and shame. In 80% of cases, the perpetrator was known to the victim. This often complicates the trauma response because of feelings of betrayal and anger related to being unable to punish the perpetrator.

### Emotion dysregulation: anger as a common behavioral manifestation

Anger, irritability, and emotional fluctuations emerged as the most consistently reported themes across all three datasets, making affective dysregulation a central theme of post-sexual trauma symptomatology. Adolescents described feeling angry and acting out of that anger, parents observed reactivity, shouting, and hitting, while professionals framed this as emotional dysregulation and mood lability. This convergence suggests that anger might be a more visible trauma response than fear or sadness, making it the primary lens through which trauma is manifested in adolescent behavior. As 87% of our sample were positive on PTSD screening, anger can also be considered a core issue of PTSD (Franklin et al., 2002). Clinically, the findings support conceptualizing anger as a trauma-related response, which has implications for the use of trauma-informed approaches in assessment and intervention. Furthermore, as there was a preponderance of female adolescents in our sample, our findings may be supported by an anger typology depicting four types of anger following sexual abuse ranging from non-productive, self-castigating behavior to empowering, righteous anger that may help survivors prevent further abuse and advocate for abused children (e.g., concern for younger siblings in our study; Thomas et al., 2012). As overt violence was not prominently reflected in our codes, we could cautiously consider this as adaptive anger, which entails a clear statement of the perceived offense and violation of personal rights (Thomas et al., 1998; De Rivera, 2006).

### Emotion dysregulation: severe forms of maladaptive behavioral manifestation and disturbed internal worldview

Adolescents and professionals both reported moderate to high levels of self-harm, avoidance, and other internalizing issues. Our findings support earlier studies reporting that self-harming behavior in sexually abused adolescents with post-traumatic symptoms is driven by negative cognition about themselves and the world rather than being solely an impulsive response to intense emotions (Choi and Song, 2025). Parents occasionally reported these behaviors, often labeling them as stubbornness or misbehavior.

Although parents intended to provide reassurance, which is a positive strategy, it was often “parent-centric,” focusing on the parent’s happiness or their perceived need to provide material support, rather than “child-centric,” focusing on the child’s internal feelings of shame or fear.

### Emotion dysregulation: disturbed external worldview

Adolescents shared fear and betrayal specific to the perpetrator, particularly when the perpetrator was known. However, parents rarely articulated relational rupture. In contrast, professionals identified attachment insecurity across relationships, erosion of trust in caregivers, and persistent fear of abandonment. This mismatch suggests that relational trauma is largely implicit for adolescents and invisible to parents. Hence, relational sequelae of abuse may remain unaddressed unless explicitly explored, increasing the risk for long-term attachment and interpersonal difficulties.

### Emotion dysregulation: disturbed internal worldview

Shame-based self-concept issues and self-harm were prevalent in professional narratives but minimally acknowledged by parents. Adolescents expressed self-directed anger or self-harm wishes, but without labeling these as shame or guilt. Parents largely failed to recognize internal self-concept disruptions. This indicates that shame may operate as a covert trauma outcome, requiring clinical inference and exploration rather than self-report. Therefore, beyond the immediate psychological impact of sexual violence, internalized shame represents a silent yet deeply entrenched consequence that frequently escapes clinical detection despite its pivotal role in long-term recovery. A Swiss epidemiological cohort study indicated that difficulties in ER functioned as a central mechanism linking adverse childhood experiences to poorer psychosocial functioning, including reduced social support, impaired interpersonal functioning, and increased use of mental health services in late adolescence and young adulthood (Brodbeck et al., 2022). The study further identified maladaptive social information processing as a partial mediator, which aligns with adolescents’ reported difficulties in interpreting social cues, contributing to relational disengagement, reduced perceived support, and emotion dysregulation.

### Gender dynamics in experience and expression of emotions and emotion dysregulation

In the qualitative phase, female survivors demonstrated greater exposure to intrafamilial abuse, penetrative assault, and repeated victimization occurring over extended periods than male survivors. Male participants reported predominantly extrafamilial, non-penetrative sexual abuse occurring in school or neighborhood settings. Although male adolescents experienced internalized emotions similar to those of female adolescents, their anger toward the perpetrator was accompanied by feelings of helplessness and powerlessness during the abusive experience, difficulty in disclosure, nervousness in social situations, and reduced confidence. By contrast, female survivors reported a broader and more intense range of emotional responses across both the affective and anxiety spectrum, including fear, sadness, anxiety, irritability, mood fluctuations, frequent crying spells, social withdrawal, reduced interest in daily activities, self-blame, and guilt. Attributing the abuse to their own perceived actions or inability to resist the perpetrator perhaps resulted in self-blame, feelings of shame, and concerns related to social stigma, family reputation, future marriage prospects, and identity. Some participants also reported thoughts of self-harm and intense emotional dysregulation, indicating severe psychological distress and maladaptive coping. This finding is supported by literature highlighting that girls exhibit higher levels of internalizing symptoms and greater ER difficulties than boys in general populations and clinically referred youth (Levkovich et al., 2025). Although the qualitative findings reflected gender differences in the experience and expression of emotions and emotion dysregulation, the quantitative findings indicated no significant difference in PTSD, and the majority of participants scored above the cutoff on PTSD (Bicanic et al., 2013; Vagni et al., 2022). However, higher mean depression scores in female adolescents may be associated with higher rates of repeated abuse and intrafamilial victimization within the female sample. Depressive symptoms may have been associated with ER difficulties, particularly among adolescent girls (Levkovich et al., 2025).

### Parents’ reactions and management of adolescents’ emotions

Although findings from an existential-phenomenological study exploring parents’ experiences focused on the significance of psychological help provided to their sexually abused adolescents, other studies highlighted the importance of parents’ own meaning-making processes regarding their perceived failure in their protective role (McElvaney and Nixon, 2020; Vladimir and Robertson, 2020). Our study provided a crucial qualitative foundation from a developing country by adding information on parents’ management of adolescents’ emotions and family situations following sexual abuse. Supportive strategies included providing space for the adolescents and allowing them to calm down, active listening and guidance, and constructive distraction (e.g., typing and knitting classes). Environmental control strategies included maintaining social distance from relatives and neighbors, strict vigilance regarding visitors to avoid triggers, moving to a different location, and dropping out of school (to prevent harm during dissociative fainting or running away from home). Maladaptive responses included physical punishment (by fathers/uncles), taunting, and blaming (by mothers/aunts) in response to adolescents’ anger toward those around them. Our findings were similar to those of previous qualitative studies on parents of sexually abused children; however, parental blaming was not a frequent code, unlike in earlier studies. Rather, adolescents’ self-blame was more common than parents’ self-blame for the occurrence of the incident (Melville et al., 2014; Mauny et al., 2023; Bayrak and Uzun, 2025). Future researchers may focus on the phenomenology of parental blaming, reactivation of parents’ traumatic past experiences, and how these factors affect the parent–child relationship, as such work could contribute valuable insights to the literature (Mauny et al., 2023). Although we did not elicit data on whether parental blaming was related to the perpetrator’s age, as reported previously, we found that most parents did not blame their adolescents, suggesting parental acceptance and support (Walsh et al., 2012).

## Summary

The findings from qualitative and quantitative data converged and supplemented each other. The findings also reinforced the value of triangulated qualitative approaches with multiple informants and corroboration through quantitative data.

Themes related to the experience and expression of post-sexual abuse emotions among adolescents showed high agreement across stakeholders regarding externally visible behavioral manifestations of emotions, such as anger, withdrawal, and anxiety symptoms. However, themes related to more internal emotional experiences (e.g., shame, guilt, and relational schemas) and process-oriented emotional responses, as conceptualized within the SPAARS model (associative—automatic emotion processing; schematic—processing requiring effortful appraisal; and propositional systems—which indirectly operate via connections with the schematic and associative levels), showed lower agreement and emerged primarily in adolescents’ narratives and professionals’ interpretations. These internalized emotional experiences were also associated with deliberate self-harm among adolescents. In contexts where stigma, fear of blame, and cultural silence surrounding sexual topics delay disclosure, adolescents are frequently left to manage intense feelings of shame, fear, anger, numbness, and mood fluctuations without adequate personal (family and peer) and professional support. This finding aligns with an earlier quantitative study (Fernández-García et al., 2023, which reported limited access to emotional regulation strategies among sexually victimized adolescents in residential care. However, unlike their findings regarding lack of emotional clarity and non-acceptance of emotional responses, adolescents in our study demonstrated relatively clear expression and acceptance of their emotions. This difference may be attributable to the qualitative nature of this study.

Limited access to professional support for adaptive emotional regulation strategies, along with parents’ relatively limited focus on understanding adolescents’ internal emotional turmoil, may contribute to self-injury functioning as an attempt to relieve emotional tension, regulate intrusive memories, or regain a sense of control.

## Study limitations

As the study was gender-skewed, the findings on emotional dysregulation and its behavioral manifestation may differ substantially among male adolescents and therefore have limited generalizability. Moreover, the quantitative sample size was small, limiting the statistical power and the conclusiveness of the findings. The adolescents included in this study were recruited from specific child-care shelter homes; therefore, patterns of emotional dysregulation may differ among sexually abused adolescents living with their families. Additionally, not all samples consisted of adolescents–caregiver dyads, and parents’ reports in non-dyad cases may have influenced the findings. As adolescents’ relationships with their families and the support received from family members play a significant role in emotional regulation, these aspects could have been explored in more detail to better understand their mediating or moderating effects following sexual abuse.

## Implications and future directions

Although the sample size of this study was based on the principles of saturation and pragmatic considerations in line with relevant literature (Britten, 1995; Morse, 2000; Ritchie and Lewis, 2003; Hammersley, 2015; Malterud et al., 2016), future studies may include a greater number of parent–adolescent dyads to enhance data richness, information power, and overall research quality. Emotional regulation may also be compared between adolescents involved in consensual romantic relationships and those who experienced sexual abuse without consent. Furthermore, emotional regulation could be explored among pregnant and non-pregnant adolescents, as well as among those receiving counseling or psychotherapy compared with those not receiving psychological support.

## Conclusion

The findings from this embedded mixed-methods study with multiple informants support the SPAARS model. Specifically, the findings confirmed that experiences of sexual abuse predispose adolescents to emotional regulation difficulties and increase the risk of IMHPs such as depression, anxiety, and PTSD. This study also supports the World Health Organization’s statement emphasizing that the heightened risk of internalizing mental health disorders are among the leading causes of illness and disability among adolescents. Furthermore, this study contributes to the literature by providing insights into parents’ management of adolescents’ extreme emotions and family situations following sexual abuse. In contexts where help-seeking was often constrained, self-harm emerged as a particularly salient and concerning manifestation of emotional dysregulation among sexually abused adolescents in residential care.

## Data Availability

The raw data supporting the conclusions of this article will be made available by the authors, without undue reservation.
