# Correction: Developmental dynamics of emotional dysregulation among sexually abused adolescents: an embedded mixed-methods study

**DOI:** 10.3389/fpsyg.2026.1949811

**Published:** 2026-08-17

**Authors:** Shreya Kulshreshtha, Sujata Satapathy, Renu Sharma, Rajesh Sagar, Reeta Mahey

**Affiliations:** 1 Department of Psychiatry, All India Institute of Medical Sciences New Delhi, New Delhi, India; 2 Department of Obstetrics and Gynaecology, All India Institute of Medical Sciences New Delhi, New Delhi, India

**Keywords:** adolescent sexual abuse, developmental emotional regulation, embedded mixed method, emotional dysregulation, mixed methods study, post traumatic stress disorder, Depression, anxiety

Author [Shreya Kulshreshtha] was erroneously listed as corresponding author. The correct corresponding author(s) is [Sujata Satapathy].

The original version of this article has been updated.

